# An Unsupervised Data-Driven Model to Classify Gait Patterns in Children with Cerebral Palsy

**DOI:** 10.3390/jcm9051432

**Published:** 2020-05-12

**Authors:** Julie Choisne, Nicolas Fourrier, Geoffrey Handsfield, Nada Signal, Denise Taylor, Nichola Wilson, Susan Stott, Thor F. Besier

**Affiliations:** 1 Auckland Bioengineering Institute, University of Auckland, 70 Symonds street, Auckland 1010, New Zealand; g.handsfield@auckland.ac.nz (G.H.); t.besier@auckland.ac.nz (T.F.B.); 2 Léonard de Vinci Pôle Universitaire, Research Center, 92 916 Paris La Défense, France; n.fourrier@gmail.com; 3 Health and Rehabilitation Research Institute, Auckland University of Technology, North Shore Campus, Private Bag 92006, Auckland 1142, New Zealand; nada.signal@aut.ac.nz (N.S.); denise.taylor@aut.ac.nz (D.T.); 4 Starship Children’s Hospital, Auckland District Health Board, 2 park road, Auckland 1023, New Zealand; n.wilson@auckland.ac.nz (N.W.); s.stott@auckland.ac.nz (S.S.)

**Keywords:** cerebral palsy, 3D gait analysis, data-driven model, ankle foot orthosis, gait variable score

## Abstract

Ankle and foot orthoses are commonly prescribed to children with cerebral palsy (CP). It is unclear whether 3D gait analysis (3DGA) provides sufficient and reliable information for clinicians to be consistent when prescribing orthoses. Data-driven modeling can probe such questions by revealing non-intuitive relationships between variables such as 3DGA parameters and gait outcomes of orthoses use. The purpose of this study was to (1) develop a data-driven model to classify children with CP according to their gait biomechanics and (2) identify relationships between orthotics types and gait patterns. 3DGA data were acquired from walking trials of 25 typically developed children and 98 children with CP with additional prescribed orthoses. An unsupervised self-organizing map followed by k-means clustering was developed to group different gait patterns based on children’s 3DGA. Model inputs were gait variable scores (GVSs) extracted from the gait profile score, measuring root mean square differences from TD children’s gait cycle. The model identified five pathological gait patterns with statistical differences in GVSs. Only 43% of children improved their gait pattern when wearing an orthosis. Orthotics prescriptions were variable even in children with similar gait patterns. This study suggests that quantitative data-driven approaches may provide more clarity and specificity to support orthotics prescription.

## 1. Introduction

Ankle–foot orthoses (AFOs) and foot orthoses are commonly prescribed to help children with cerebral palsy (CP) maintain independent mobility [1,2]. Orthoses may provide a stable base of support, improve gait mechanics, and reduce metabolic cost. Over the years, a wide variety of orthotic designs have been developed to address the different gait deviations seen in children with CP [1,3]. Common orthotic designs are the solid/rigid AFO, posterior leaf spring (PLS) AFO, hinged AFO, and the ground reaction AFO (GRAFO) [4]. Each orthotic design targets different aspects of the gait pattern; for example, the solid/rigid AFO primarily aims to restrict ankle plantarflexion and dorsiflexion to enable heel strike in the stance phase and toe clearance in the swing phase, whereas the PLS AFO enhances push-off power in the terminal stance by acting like a spring at the ankle [5,6]. 3D gait analysis (3DGA) is used as part of clinical care for children with CP; gait data are typically collected for both barefoot walking and walking with the orthosis. This provides information about the effectiveness of the orthosis in positively changing the gait pattern and supports clinicians’ decisions around changes in orthotic prescription [7]. 3DGA measurements are accurate, but interpretation of this complex information can be challenging [8,9].

Previous researchers have attempted to summarize 3DGA data into a single measure of “quality” of the gait pattern. Schwartz and colleagues [10] developed the Gait Deviation Index (GDI), which quantifies the differences in kinematics between a patient’s gait cycle and a reference dataset. Because the GDI is a single measure of the gait deviation between a child with CP and typically developing (TD) children, this index does not show the levels at which the gait deviations are present. To address this, Baker and colleagues [8] developed the Movement Analysis Profile (MAP), which represents the RMS difference across time between a child with CP’s gait cycle and an average TD child’s gait cycle, calculated for a single gait variable rather than the entire gait vector. They referred to it as the gait variable score (GVS) for the nine relevant kinematic variables. However, the direction of the gait deviation is not captured by this score. While such measures can quantify overall gait biomechanics, monitor progress, or evaluate the outcome of an intervention, it is unclear whether these parameters can be used to inform orthotic prescription.

Data-driven modeling can reveal non-intuitive relationships between variables, such as between 3DGA parameters and gait outcomes with orthotics in children with CP. This method can process large quantities of data without exclusively relying on a priori knowledge of predictive variables [11]. For example, previous studies were able to predict which children with CP would benefit from rectus femoris muscle transfer surgery or hamstring-lengthening surgery in crouch gait with 88% and 73% accuracy, respectively, by combining 3DGA and musculoskeletal modeling [12,13]. More recently, researchers [14] developed a statistical orthosis selection model using a supervised learning model, i.e., a random forest algorithm. Model development was carried out using retrospective data from gait analysis performed on children with CP who wore one of five orthotic designs bilaterally. Their model recommended a specific AFO design or no AFO depending on the GDI improvement seen in their training dataset. Their model consisted of five independent random forest ensembles with different characteristics and features drawn from medical history, physical examination measures, and kinematic data, making it difficult to evaluate the relative importance of each feature. Their model agreed with the prescription only 14% of the time for limbs with an orthosis, and for 56% of limbs without an orthosis, the model agreed that no orthosis was expected to provide benefit. By using a random forest algorithm (one per selected AFO), the researchers utilized between four and nine features (depending on each forest) and showed promising predictive performance accuracies between 67% and 82%, with prediction errors between 5.1 and 6.2 GDI points. These high prediction errors could be due to poor AFO prescription efficacy in the current standard of care. The model did not provide a biomechanical rationale for the selection of an AFO, which was dependent on the features selected for each forest from the random forest algorithm. The main limitations with this study were the characteristics of the random forest algorithm, which is a supervised data-driven model. Supervised models require a priori knowledge to relate data input to their associated output, which requires manual data labeling and therefore introduces human bias. On the other hand, unsupervised learning models (such as self-organizing maps) aim to uncover underlying similarities that could not be revealed otherwise.

The present study used a self-organizing map (SOM) to (1) develop a data-driven model to classify children with CP according to their gait biomechanics and (2) identify relationships between orthosis types and the gait patterns revealed by the model.

## 2. Experimental Section

This study received ethical approval from the University of Auckland Human Participants Ethics Committee (UoA HPEC 017025) and from the Auckland District Health Board Research Review Committee in New Zealand (ADHB-RRC 7202_20160718). This study was a minimal risk observational study as it involved the retrospective analysis of de-identified clinical data which were not linked back to any individual; therefore, patients did not provide written informed consent for this study.

### 2.1. Participants

Kinematic data from children with CP collected as part of standard clinical care at the AUT Motion Analysis Laboratory, Auckland, New Zealand, were retrospectively analyzed. The AUT Motion Analysis Laboratory used a Qualisys motion capture system (Qualisys AB, Göteborg, Sweden) with nine cameras and a modified Helen Hayes marker set [15]. Children were asked to walk (1) barefoot and (2) with their prescribed orthosis at their self-selected speed on a 10 m runway, with mean gait data derived from a minimum of five representative gait trials for each condition. Inter and intra-rater reliability was routinely assessed in the clinic, with marker placement accuracy quality checked prior to data collection for each child in each condition. A total of 98 children with CP, GMFCS levels I to III (age: 10.3 ± 3.3 years), were included in the analysis. Selected children had already been prescribed either unilateral orthoses (*n* = 27), bilateral orthoses (*n* = 56), or no orthoses (*n* = 15) based on the decisions of a multi-disciplinary team in consultation with the child and their guardian [1]. Exclusion criteria were any history of orthopedic surgery or botulinum toxin A injections within the past 6 months. We also included barefoot walking kinematic data from 25 typically developing (TD) children as a control reference dataset. Given that many children with CP have asymmetric involvement, we decided to treat the two lower limbs as independent variables, including the uninvolved unilateral limb that was not prescribed an orthosis.

### 2.2. Model Input

The model input data consisted of the gait variable scores (GVSs) extracted from 3DGA performed walking barefoot [8]. The GVS were determined as follows: for each joint, we calculated the RMS difference across time for one gait cycle (51 values, representing a gait cycle from 0 to 100% every 2%) between a child with CP’s gait cycle and the average gait cycle from our TD children reference dataset. For the hip, knee, and ankle range of motion in the sagittal plane, we differentiated between the GVS for flexion (when the patient displayed higher flexion angles than the TD reference dataset) and the GVS for extension (when the patient displayed higher extension angles than the TD reference dataset). Foot progression angle was also differentiated in the same way (a score for greater outward angles and a score for greater inward angles). Therefore, a total of 13 GVSs were calculated: pelvic tilt, pelvic obliquity, pelvic rotation, hip flexion, hip extension, hip abd/adduction, hip rotation, knee flexion, knee extension, ankle dorsiflexion, ankle plantar flexion, foot progression inward, and foot progression outward.

### 2.3. Data-Driven Model

**Figure 1:**
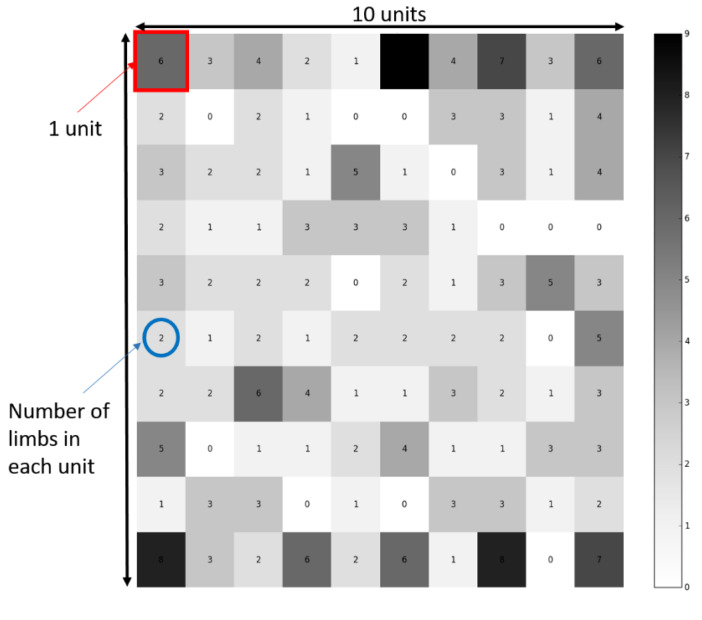
The self-organizing map (SOM) grouped together participants with similar inputs. The map was a 10 × 10 square units; each limb was localized in one of the 100 square units. A square unit could have multiple limbs (the number in each unit is displayed in the square unit). Limbs grouped together in the same unit on the map had similar inputs. This figure represents the distribution of each participant limb in the map, with white being no limbs in the unit and black being nine participants’ limbs associated to the unit.

The model was developed in Python version 3.4 using an in-house code and the SOMPY library [16]. The model consisted of a self-organizing map (SOM), which is an unsupervised data-driven model that represents high-dimensional data in a two-dimensional (2D) map representation by grouping participants together based on shared features/similar input (Figure 1) [17]. SOM is a type of artificial neural network that uses a neighborhood function to preserve topological properties of the input space, giving an insight into the topographical relationships of data [17]. SOMs differ from other artificial neural networks (NN) as SOMs apply competitive learning as opposed to error-correction learning NN. The concept of the SOM is very similar to other clustering algorithms in the sense that it computes distance between reference elements (centroid for k-means, each node of the grid in the SOM case), but reduces the requirement for a priori knowledge and allows for dynamic updates while maintaining previously discovered patterns. SOM produces a distorted space where inputs similar to each other are grouped close to each other on the 2D grid. Each limb is associated in one unit, where multiple limbs can be placed in the same unit (Figure 1; number in each unit represents number of limbs associated in each unit). Once produced, the SOM space is frozen and each limb remains within its unit. In other words, the model groups together limbs with similarities based on their input data. The size of the 2D map was set to be a 10 × 10 grid of units (Figure 1).

The notion of clusters in SOM is an interpretation that can be added only after the space is computed using the SOM and a subsequent k-means algorithm is applied to form these clusters. It is important to note that such an approach allows the best neighbors to be found for a given sample. In the present study, after the SOM allocated each limb into a unit on the grid based on neighborhood similarities (Figure 1), a k-means clustering algorithm was used to partition the resulting SOM map into distinct clusters, in which each observation belongs to the cluster with the nearest mean [18]. A sensitivity analysis was performed to determine the number of clusters needed for this dataset.

### 2.4. Statistical Analysis

A statistical analysis was performed in order to understand whether and how groups resulting from the SOM + k-means clustering algorithm were different. A Levene test of homogeneity of variance and a normality test were completed to ensure the validity of assumptions needed to proceed with a one-way ANOVA test. Each cluster group (independent variable) was compared for each of the 13 GVSs (model input for each limb), i.e., the dependent variable, with a Bonferroni correction (*p* = 0.008) pairwise comparison based on the number of clusters.

### 2.5. Relationships between AFO Types and Gait Pattern

**Figure 2:**
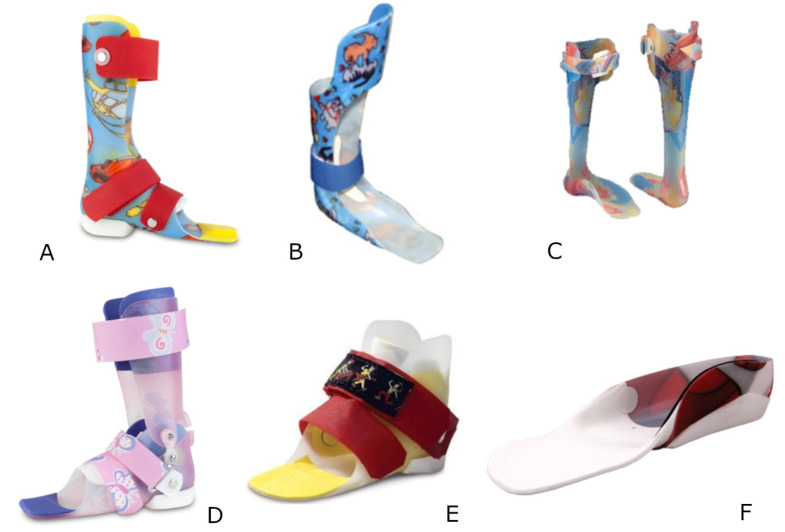
Example of (**A**) solid Ankle Foot Orthosis (AFO), (**B**) ground reaction AFO (GRAFO), (**C**) posterior leaf spring AFO (PLS AFO), (**D**) hinged AFO, € supra-malleolar orthosis, and (**F**) sport dynamic orthosis used in this study.

In children with CP, 3DGA data collected when wearing their prescribed orthosis were also evaluated. In New Zealand, all orthoses are custom-molded to the shape of the foot with posting as needed to align the forefoot and hind foot neutrally and set the floor to shank angle to neutral. The posting on the Ankle Foot Orthosis (AFO is on the outside of the AFO, i.e., the forefoot and hind foot are set as close to neutral as possible and the floor to shank angle is also set as close to neutral as possible. The exterior of the AFO is posted to correct any residual misalignment. Orthoses prescribed in this cohort were solid AFOs, ground reaction AFO (GRAFO), posterior leaf spring (PLS) AFOs, hinged AFOs, supra-malleolar orthosis (SMOs), and sport dynamic FOs. Solid AFOs and GRAFOs do not allow ankle dorsiflexion. Posterior leaf spring (PLS) AFOs and hinged AFOs both block plantarflexion at the ankle. PLS AFOs restrict ankle dorsiflexion to 5–10 degrees, whereas the hinged AFO allows free ankle dorsiflexion within the existing ankle range. SMOs and sport dynamic FOs do not constrain the ankle (Figure 2). For each trial, a gait profile score (GPS) was calculated as the RMS average of all the GVSs for a particular limb [8]. The difference in GPSs between barefoot and with orthosis was calculated for each limbs (ΔGPS = GPS barefoot – GPS orthosis). If the difference (ΔGPS) was negative, the orthosis did not improve gait kinematics; if the difference was positive, the orthosis improved gait kinematics (+ΔGPS). Two variables were derived from ΔGPS: (1) the average GPS difference (Average ΔGPS = sum(ΔGPS)/#limbs) and (2) the average GPS improvement (Average +ΔGPS = sum (+ΔGPS)/#limbs with improved GPS).

## 3. Results

### 3.1. Data-Driven Model

**Figure 3:**
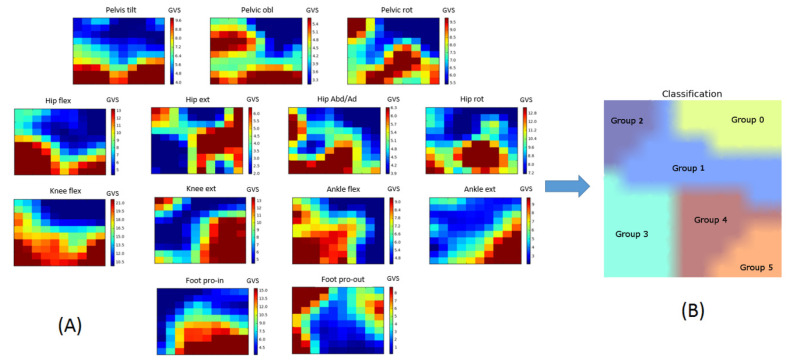
Self-organizing map (SOM) outcome for each gait variable score (GVS) (**A**) and overall SOM map (**B**). Limbs placed close together on the maps had similar gait patterns. In subfigure (**A**), the color scale represents the average GVS of limbs in each unit; blue represents a low GVS and red represents a high GVS. Limbs with low GVSs were usually grouped together, and the same was observed for limbs with high GVSs. Subfigure (**B**) represents the group results after the k-means algorithm was applied. The following abbreviations are used: obl = obliquity, rot = int/ext rotation, flex = flexion, ext = extension, abd = abduction, add = adduction, pro-in = progression inward, and pro-out = progression outward.

The sensitivity analysis for the k-means clustering algorithm indicated that the optimal number of clusters for our dataset was six distinct groups (Figure 3B). Group 0 (*n* = 50) was composed of the limbs of typically developing children, while Groups 1 to 5 consisted exclusively of the limbs of children with CP (refer to supplementary material for details). The GVS distribution for each gait feature across the self-organizing map is displayed in Figure 3A. For each score, the SOM algorithm grouped together the limbs of children with CP with high scores away from limbs with low scores.

**Figure 4:**
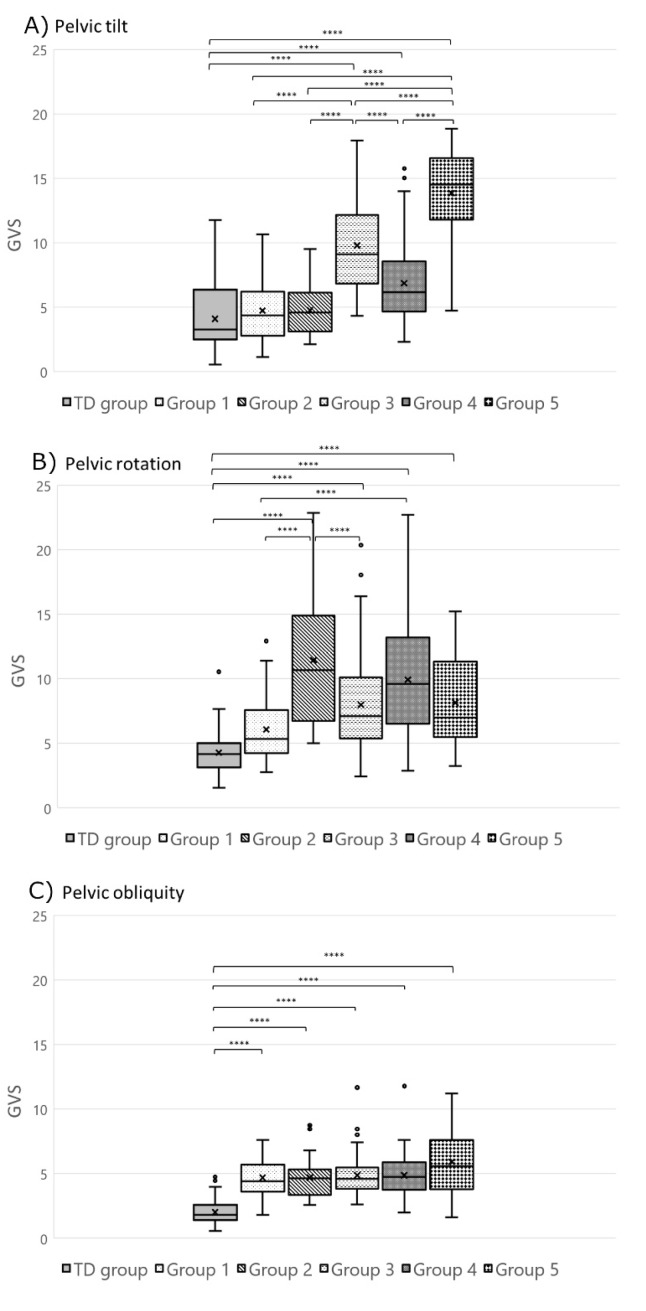
Box plot representation of the gait variable score (GVS) distribution at the pelvis (**A**) tilt, (**B**) rotation, and (**C**) obliquity) for each group. In the box plots, the boundary of the box closest to zero indicates the 25th percentile, a black line within the box marks the median, the cross inside the box marks the mean, and the boundary of the box farthest from zero indicates the 75th percentile. Whiskers above and below the box indicate the 10th and 90th percentiles. Points above and below the whiskers indicate outliers outside the 10th and 90th percentiles. TD group: typically developing children (control group). Statistical differences between groups are noted with **** *p* < 0.0001.

**Figure 5:**
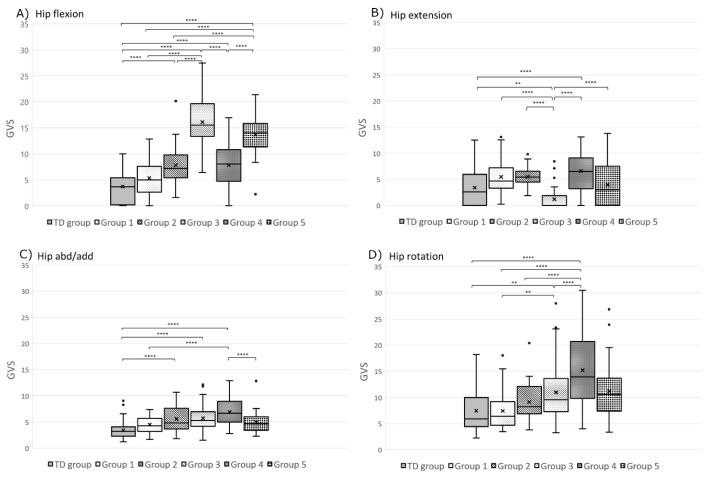
Box plot representation of the gait variable score (GVS) distribution at the hip (**A**) flexion, (**B**) extension, (**C**) abd/adduction, and (**D**) int/ext rotation for each group. In the box plots, the boundary of the box closest to zero indicates the 25th percentile, a black line within the box marks the median, the cross inside the box marks the mean, and the boundary of the box farthest from zero indicates the 75th percentile. Whiskers above and below the box indicate the 10th and 90th percentiles. Points above and below the whiskers indicate outliers outside the 10th and 90th percentiles. TD group: typically developing children (control group). Statistical differences between groups are noted with **** *p* < 0.0001, ** *p* < 0.01.

**Figure 6:**
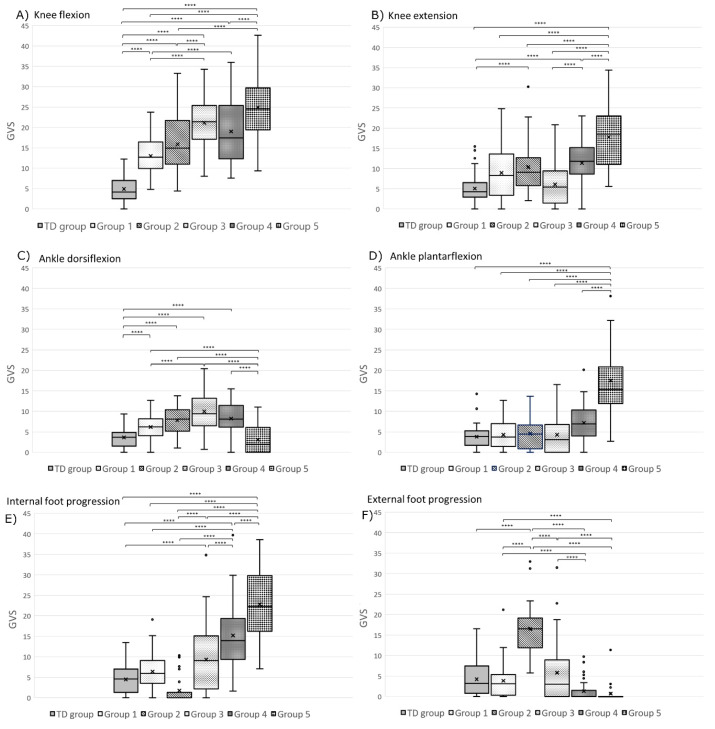
Box plot representation of the gait variable score (GVS) distribution at the knee (**A**) flexion and (**B**) extension), ankle (**C**) dorsiflexion and (**D**) plantar flexion, and for the foot progression angle (**E**) internal and (**F**) external for each group. In the box plots, the boundary of the box closest to zero indicates the 25th percentile, a black line within the box marks the median, the cross inside the box marks the mean, and the boundary of the box farthest from zero indicates the 75th percentile. Whiskers above and below the box indicate the 10th and 90th percentiles. Points above and below the whiskers indicate outliers outside the 10th and 90th percentiles. TD group: typically developing children (control group). Statistical differences between groups are noted with **** *p* < 0.0001.

The Levene test of homogeneity of variance and normality test indicated that population variances in each group were equal and normally distributed. The one-way ANOVA showed a statistically significant difference between groups for each dependent variable (GVS score). Pairwise comparisons of the gait variable score (GVS) distribution are displayed for the pelvis (Figure 4), hip (Figure 5), knee (Figure 6), and foot and ankle (Figure 6) for each group using box plot representation. Statistical differences between groups were as follows.

- Group 1: Statistical differences between Group 1 (*n* = 49) and TD children group were found for three GVS features; pelvic obliquity (*p* < 0.0001), knee flexion (*p* < 0.0001), and ankle dorsiflexion (*p* = 0.003) (Figure 4 and Figure 6).
- Group 2: Limbs classified in Group 2 (*n* = 24) had a statistically significantly greater external foot progression angle GVS throughout the gait cycle compared to all the other groups (Figure 6). Group 2 limbs had greater GVSs than the TD children group for the following joint motions: pelvic rotation (*p* < 0.0001), pelvic obliquity (*p* < 0.0001), hip flexion (*p* < 0.0001), hip add/abd (*p* = 0.001), knee flexion (*p* < 0.0001), knee extension (*p* = 0.002), and ankle dorsiflexion (*p* < 0.0001) (Figure 4, Figure 5 and Figure 6).
- Group 3: Limbs in Group 3 (*n* = 56) had statistically significantly greater hip flexion GVSs than all the other groups (Figure 5). They also had a higher pelvic tilt (*p* < 0.0001), pelvic obliquity (*p* < 0.0001), pelvic rotation (*p* < 0.0001), hip extension (*p* = 0.006), hip add/abd (*p* < 0.0001), hip rotation (*p* = 0.008), knee flexion (*p* < 0.0001), ankle dorsiflexion (*p* < 0.0001), and internal foot progression (*p* = 0.002) than the limbs of TD children (Figure 4, Figure 5 and Figure 6).
- Group 4: Limbs in Group 4 (*n* = 39) displayed statistically significantly greater hip rotation GVSs than the other groups (Figure 5). Limbs in this group also had a greater GVS than TD children in all gait features (*p* < 0.0001) except for ankle plantar flexion (*p* = 0.009) and external foot progression (*p* = 0.131) (Figure 4, Figure 5 and Figure 6).
- Group 5: Limbs classified in Group 5 (*n* = 28) had statistically significantly greater pelvic tilt, knee extension, ankle plantar flexion, and internal foot progression GVSs than all the other groups (*p* < 0.0001) (Figure 4 and Figure 6). Group 5 also had greater pelvic obliquity and rotation (*p* < 0.0001) and hip and knee flexion (*p* < 0.0001) than TD children (Figure 4, Figure 5 and Figure 6).

**Figure 7:**
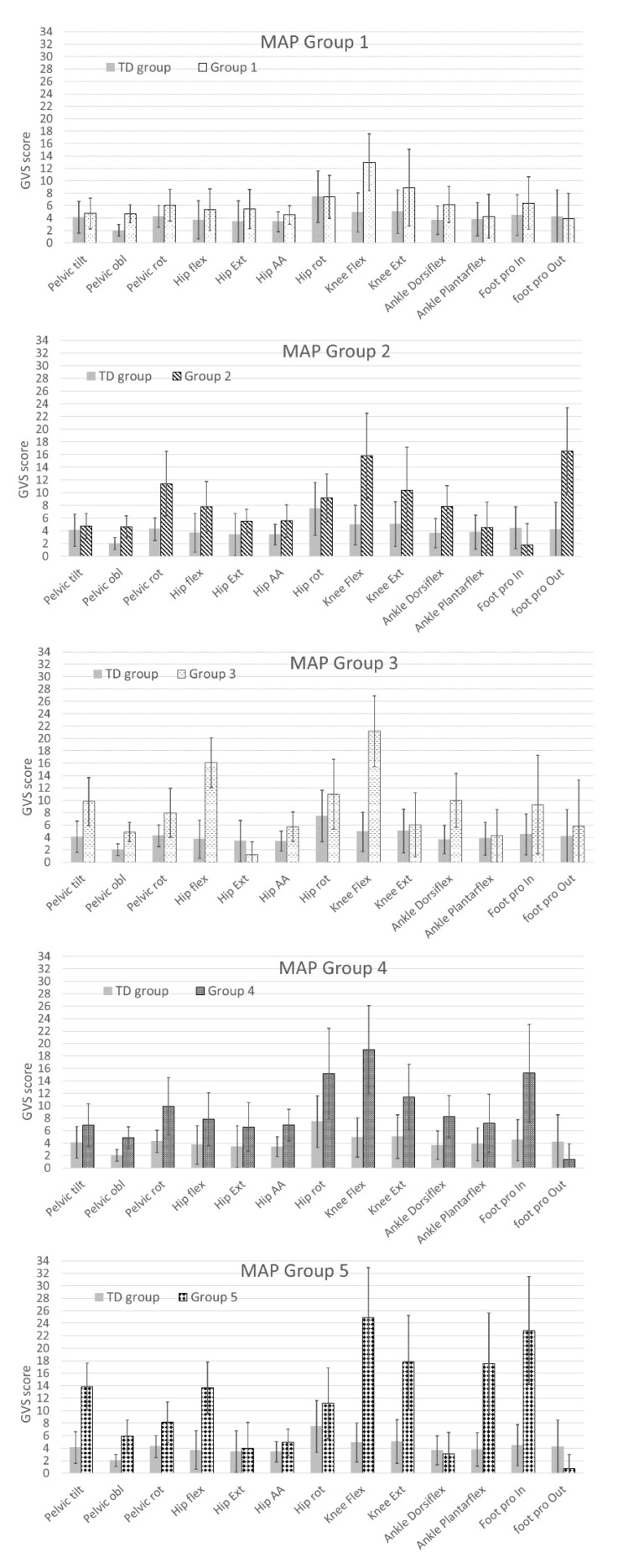
Groups 1 to 5 movement analysis profiles (MAPs) [8]. Each column corresponds to one of the kinematic variables (13 GVS inputs to the model). The column height represents the average GVSs of children with cerebral palsy associated with each group (pattern) and the average GVSs of typically developing children (grey). The following abbreviations are used: obl = obliquity, rot = int/ext rotation, flex = flexion, ext = extension, AA = abduction/adduction, Dorsiflex = dorsiflexion, Plantarflex = plantar flexion, pro-in = progression inward, and pro-out = progression outward.

A movement analysis profile (MAP) representation [8] for each group is presented in Figure 7, comparing the GVSs of Group 1 to 5 with the typically developed children’s GVSs. For Group 1, we observed that GVSs of the children with CP included in this group had similar GVSs as the TD children, except for pelvic obliquity and knee flexion (Figure 7; MAP Group 1). Children with CP included in Group 2 had a higher score than TD children for all variables except for inward foot progression angle (Figure 7; MAP Group 2). Group 3 children with CP had higher GVSs than TD children except for hip extension GVS score (Figure 7; MAP Group 3). Children with CP in Groups 4 and 5 had higher GVSs than TD children except for outward foot progression angle (Figure 7; MAP Groups 4 and 5). However, children with CP in Group 5 had similar GVSs as TD children for hip extension, hip abduction/adduction, and ankle dorsiflexion (Figure 7; MAP Group 5).

### 3.2. Relationships between Orthosis Prescription and Gait Pattern

**Figure 8:**
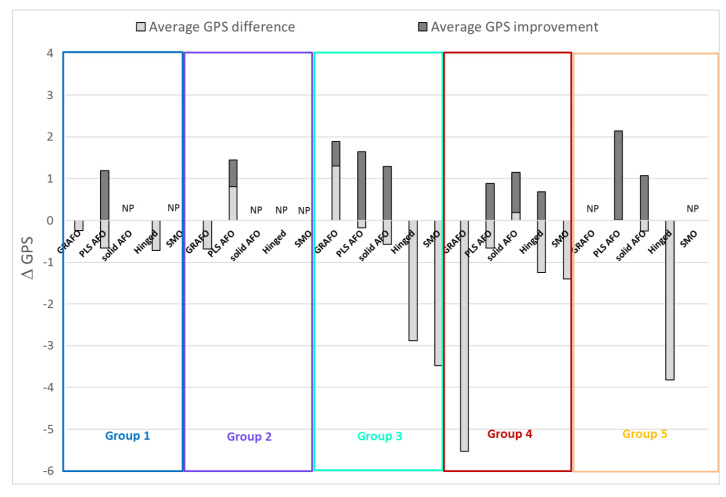
Average gait profile score (GPS) differences between walking barefoot and with the prescribed orthosis per group and per AFO. Light grey bars represent the average GPS difference (average ΔGPS = sum (ΔGPS)/#limbs) and dark grey bars represent the average GPS improvement (Average +ΔGPS = sum (+ΔGPS)/#limbs with improved GPS), i.e., when improved GPS limbs were taken into consideration. ΔGPS = (GPS barefoot - GPS orthosis), +ΔGPS is when the difference was positive (improvement OF GPS with AFO), NP: not prescribed to the group, GRAFO: ground reaction AFO, PLS AFO: posterior leaf spring AFO, SMO: supra malleolar orthosis, #: number.

Of the 196 limbs from children with CP included in the model, 139 were prescribed an orthosis (Table 1). Only 43% of the 139 limbs demonstrated an overall improvement in their GPS with orthosis use. In Group 1, 30% of the limbs showed an improved GPS with the prescribed orthosis (PLS AFO only), with an average improvement of 0.9° (14%) (Figure 8). In Group 2, 54% of the limbs showed an improved GPS with the prescribed orthosis. Most of the limbs classified in Group 2 that were prescribed a PLS AFO showed an improved gait score (71%), whereas none of the limbs classified in Group 2 that were prescribed a GRAFO showed an improved GPS score. In Group 3, 38% showed an improved GPS with the prescribed AFO (GRAFO, PLS AFO, or solid AFO). Limbs in Group 3 had the greatest diversity in the design of their prescribed orthoses; of this group, 48% were prescribed PLS AFO, 17% no orthosis, 15% a solid AFO, 10% GRAFO, and 10% others (including hinged, SMO, and sport dynamic FO). In Group 4, 38% showed an improved GPS with the prescribed orthosis. PLS AFO was the most prescribed AFO for Group 4, but only improved GPS for 35% of the limbs. A solid AFO improved the GPS for 50% of the limbs, with an average 1.2° (10%) GPS improvement. In Group 5, 50% of the limbs showed an improved GPS, mainly using a PLS AFO (2.1° (12%) GPS improvement) or a solid AFO (1.1° (7.5%) GPS improvement).

## 4. Discussion

The first objective of this study was to classify children with CP according to their gait biomechanics using a data-driven approach based on their GVS. We developed a self-organizing map (SOM), an unsupervised data-driven model, which provided a simple two-dimensional (2D) representation of the data (Figure 1) while retaining the topological properties of the dataset. This approach helped to understand how high-dimensional data are linked together by reducing the data dimensions to a 2D map. Principal component analysis (PCA) is another data dimension reduction algorithm that explains the maximum amount of variation in a dataset, which is well suited to identifying the dimensions that account for the largest variability. However, this algorithm will not correctly capture pathologies where the sample number is low. On the other hand, by construction, an SOM will isolate samples that present differences, disregarding the number of samples. Furthermore, an SOM offers the ability to dynamically learn, i.e., the addition of a new set of samples to the existing model will not redistribute original samples in any other way—it might refine and highlight new groups, but it will not change the dimensions of interest as could happen with PCA. While supervised models require a priori knowledge to relate data input to their associated output (e.g., support vector machines, random forest, and supervised neural networks) with a separated training set and testing set, unsupervised learning removes human bias by not requiring output data labeling; therefore, the model does not need to be trained in advance. The goal underlying the development of an unsupervised learning model was to uncover underlying similarities between children with CP, which could not be revealed otherwise. The notion of clusters in SOM is only an interpretation that can be added after the space is computed, and k-means are a candidate approach to form these clusters. It is important to note that an SOM is an intermediate layer of interpretation on top of which a clustering algorithm, k-means, was added to highlight the different clusters. It allowed the best neighbors to be found for a given sample. This is particularly relevant in such a study as one might use the clustering directly to apply a similar treatment to limbs present in a cluster. Alternatively, given a new patient, the SOM would allow clinicians to easily identify similar patients. Although our model was unsupervised (no data training), it discriminated between children with CP and typically developing children with 100% accuracy.

The second objective of this study was to identify relationships between orthosis types and the gait patterns revealed by the model. Our model was able to cluster patients’ limbs into five distinct groups with statistically significant differences between groups (Figure 4, Figure 5 and Figure 6). Statistical analysis showed very little difference between the gait patterns of limbs classified into Group 1 and TD children (Figure 7). On the SOM map (Figure 3), this group was located along the border of the TD children’s group. This means that Group 1 children have a gait pattern that is similar to TD children except for (1) excessive knee flexion, (2) reduced plantar flexion, and (3) excessive pelvic obliquity. In this group, 47% of children were not prescribed an orthosis and 47% were prescribed a PLS AFO. The GPS improved (+ΔGPS) by 1.19 points on average for 30% of the children prescribed a PLS AFO (Figure 8, dark grey bar). A previous study looking at the minimally clinically important difference (MCID) for GPS in children with CP suggested a MCID of 1.6 points, reflecting the mean difference between adjacent functional assessment questionnaire levels in children with CP [19]. This may suggest that an orthosis is not always indicated for children in this group, as their gait impairment is modest and the average GPS improvement is not considered clinically significant. Group 2 limbs were characterized by high external foot progression angle and pelvic retraction (Figure 7), which might be representative of “lever arm disease” caused by mid foot breaching and lateral tibial torsion according to the Rodda and Graham classification [20]. Another possibility is the increase in pelvic rotation GVS, which may subsequently lead to external foot progression angle. In the literature, a GRAFO can be recommended for children with rotational malalignment, lever arm dysfunction, and flexed gait knee [20,21]. However, clinicians do not usually prescribe a GRAFO in face of external tibial torsion or foot breech, which explains why clinicians prescribed GRAFOs to few children in this group, with most prescriptions being for a PLS AFO. In the two children with GRAFOs, the overall GPS did not improve (Figure 8, light grey bar), which confirmed the prescribing clinicians’ experience. Children prescribed PLS AFOs in Group 2 appeared to improve their gait, with an average GPS improvement (+ΔGPS) of 1.44 points between barefoot gait and their prescribed PLS AFO (Figure 8, dark grey bar). The gait pattern in Group 3 was characterized by a high amount of pelvic tilt motion, along with increased hip and knee flexion and ankle dorsiflexion throughout the gait cycle (Figure 7). This gait pattern is often referred to as crouch gait [20,21,22]. The recommended orthosis prescription is a GRAFO [20,22] to re-establish the integrity of the plantar flexion/knee extension (PF-KE) couple. This prescription is in agreement with our data (Figure 8, dark grey bar) where GRAFOs were prescribed to five children and improved their GPS (+ΔGPS) by an average of 1.9 points. Limbs in Group 4 had a gait pattern characterized by increased hip abd/adduction and hip internal/external rotation (Figure 7), which is representative of marked asymmetry in hemiplegia patients, such as in Type 4 hemiplegia, according to Rodda and Graham [7,20]. Recommended orthotic management includes a GRAFO, solid AFO or hinged AFO [7,20]. In our study sample, the most effective prescription for Group 4 was the solid AFO (improvement value (+ΔGPS) 1.15 pts) with PLS and hinged AFOs showing smaller GPS improvements (Figure 8, dark grey bar). Limbs clustered in group 5 had the greatest gait impairment, with increased pelvic tilt and hip flexion, reduced knee flexion in the swing phase, and increased ankle plantar flexion and internal foot progression (Figure 7). This gait pattern could be categorized as jump gait [20]. Orthotic management recommendations are based on the PF-KE couple of GRAFO, solid AFO, or hinged AFO [20]. In our study sample, half the limbs prescribed PLS AFOs and solid AFOs showed improved GPS. None of the Group 5 limbs were prescribed a GRAFO. It is notable that in this study sample, clinicians prescribed PLS AFOs to half of the total limbs (Table 1), which is not considered appropriate for all gait patterns [1,23]. After validating this gait pattern classification in a prospective study, this computational model could be used to help clinicians in their orthosis prescription decision-making.

To the best of our knowledge, no previous study has used unsupervised approaches to classify children with CP based on their gait biomechanics. One previous study developed a predictive model based on linear discriminant analysis to understand which combination of gait variables could accurately predict 88% of rectus femoris transfer surgery outcomes [12]. The same research team developed a multivariate regression model to understand which gait variables contribute to improvement in stance phase knee flexion in children with crouch gait, and were able to predict which patient would improve their knee kinematics with 70% accuracy [13]. More recently, researchers developed a random forest data-driven model to select the best AFOs for children with spastic diplegia [14]. They focused their random forest to select one out of five types of orthosis that would provide the best gait outcome for children with CP. Basically, each AFO represented a decision tree that predicted the GDI for each limb if the patient was to wear each AFO. The AFO decision tree with the best GDI outcome was therefore recommended; if none of them improved GDI, then the recommendation was no orthosis. 3DGA was performed by children with CP barefoot and wearing their prescribed AFO, which implies that all children did not wear all five AFOs, making the benefit estimation more difficult. Each AFO ensemble had different inputs, RMSE, and accuracy. In contrast, the present study concentrated on classifying children’s limbs based on their gait kinematics using each participant’s barefoot 3DGA. We then tried to understand which type of AFO would improve the overall GPS for the limbs classified in each group. Future work will concentrate on validating the model based on 3DGA obtained from prospective patients with the help of physiotherapists and orthopedic surgeons.

The relatively small sample size for our study was the main study limitation. We included data from gait analysis performed in one clinic to reduce differences in data collection protocol. We also disregarded multiple visits to avoid pseudo-replication. We also did not include clinical examination measures of children with CP such as lower limb joints’ range of motion and strength. The main reason for not including these measures as input for our model was the qualitative assessment of these measurements, which called their reliability across patients into question. 3DGA is a quantitative measurement of gait with high reproducibility and reliability in adults and children [24,25]. Another limitation was the decision to treat each leg independently; this approach did not consider the influence of inter-limb kinematics on gait pattern. Consequently, orthotic prescription was evaluated on a limb-by-limb basis, which did not consider the influence of orthotic prescription for one limb on the kinematics of the other limb in the same patient. It is also important to note that posterior leaf spring (PLS) AFOs were prescribed to 50% of the total limbs of children with CP in this study. While this cohort of retrospective clinical data was randomly selected using inclusion and exclusion criteria, it appears that PLS AFOs were prescribed more frequently in this cohort. The lack of kinetics data in this study did not allow us to differentiate GVSs for the stance/swing phase, which is a limitation that could be addressed in future work. The use of the GPS as input to the model could be considered a limitation. GPS is defined as the RMS average difference across time between a specific gait cycle and the average gait cycle from people with no gait pathology [8]. Each score represents the average differences in one joint rotation from typically developing children during the gait cycle. It is limited in its interpretation, as both temporal information and the direction of change do not feature in the data. However, if we were to input all 459 gait features in the model per limb for 246 limbs, it would considerably increase the risk of overfitting the model. Therefore, we needed a solution to reduce the number of input features. GPS is widely used in the clinical community to report improvement/non-improvement in gait analysis after surgery and to record progress over time of individual patients. That is the main reason we chose to use GPS calculation as input features for our model. Another problem, also common in orthosis research, was the lack of standardization in orthotic design [26]. As all AFOs in New Zealand are custom-fabricated, differences can exist even within the same design.

## 5. Conclusions

The present study presented an unsupervised data-driven model that classified children with CP into five statistically distinct groups according to their gait biomechanics. Future work will focus on validating the model in a prospective longitudinal study including new patients. The data collected to build this model were obtained from a single clinic; because of methodological differences in data collection between clinics, the outcomes for this modeling approach are expected to be different for different clinics.

## Figures and Tables

**Table 1:** Limb distribution in each group with the prescribed orthosis (#: number).

Prescription for Each Limb in Each Group							
# of Llimbs	No Orthosis	GRAFO	PLS AFO	Solid/Rigid AFO	Hinged AFO	Others	Total
**Group 1**	23	1	23	0	2	0	49
**Group 2**	8	2	14	0	0	0	24
**Group 3**	13	5	25	8	1	4	56
**Group 4**	11	1	17	6	3	1	39
**Group 5**	2	0	18	7	1	0	28
**Total**	57	9	97	21	7	5	196

## Supplementary Materials

The following are available online at https://www.mdpi.com/2077-0383/9/5/1432/s1. The following Excel file contains all the relevant input and output of the model. Result_0 sheet: Model input and output for each child with CP; Control sheet: Model input and output for typically developed children; patient info sheet: Children with CP clinical and demographic information; Group1 sheet: Model output for limbs clustered in group 1; Group2 sheet: Model output for limbs clustered in group 2; Group3 sheet: Model output for limbs clustered in group 3; Group4 sheet: Model output for limbs clustered in group 4; Group5 sheet: Model output for limbs clustered in group 5; statistics sheet: Statistical results; AFO sheet: Information about each patient’s prescribed AFO and GPS barefoot and with prescribed AFO.
